# Healthcare Professionals’ Perceptions of Anhedonia in Major Depressive Disorder and the Possibilities of Episodic Future Thinking Training: A Qualitative Study in a Tertiary Care Hospital

**DOI:** 10.3390/nursrep15110384

**Published:** 2025-10-28

**Authors:** Minghao Pan, Huijing Zou, Dan Luo, Xiao Qin Wang, Qian Liu, Meiyu Shen, Xiaofen Li, Xuan Gong, Bing Xiang Yang

**Affiliations:** 1 Department of Psychiatry, Renmin Hospital of Wuhan University, Wuhan 430060, China; 2022103050009@whu.edu.cn (M.P.);; 2 Center for Wise Information Technology of Mental Health Nursing Research, School of Nursing, Wuhan University, Wuhan 430060, China; 3 Research Center for Lifespan Health, School of Nursing, Wuhan University, 115 Donghu Road, Wuhan 430060, China

**Keywords:** major depressive disorder, anhedonia, episodic future thinking, psychological interventions, healthcare professionals, qualitative research

## Abstract

**Introduction**: Major depressive disorder (MDD) is a mental disorder with a high prevalence rate and a high recurrence rate. Therefore, identifying and intervening in the core symptoms of MDD patients is of great significance. Anhedonia is manifested as an individual losing interest in activities or experiencing a significant decrease in the sense of pleasure, which is one of the two core symptoms of MDD. Episodic Future Thinking (EFT) training refers to the process of stimulating individuals’ hope for positive future scenarios and encouraging them to take purposeful actions, which may have an effect in alleviating anhedonia. However, the perception of anhedonia of MDD patients among Chinese healthcare professionals is still unclear, and there has been no exploration of the views of healthcare professionals regarding the implementation of EFT training for MDD patients led by psychiatric nurses in a clinical setting. **Aim**: This study aimed to understand the attention paid by Chinese healthcare professionals to the symptom of anhedonia in patients with MDD, as well as their previous coping strategies. This study further explored the views of healthcare professionals regarding the implementation of EFT training for MDD patients led by psychiatric nurses with a psychological therapist certificate in China, as well as suggestions for future implementation. **Methods**: This qualitative descriptive study adopted a phenomenological approach. Using purposive sampling, 15 healthcare professionals (psychiatrists, psychiatric nurses and psychological counselors) were recruited from the psychiatry department of a public tertiary hospital in Wuhan, Hubei Province, China. Using the NVivo 12 Plus software, the semi-structured interviews and analyses were conducted by applying Colaizzi’s seven-step phenomenological method. Rigor was ensured through checks of credibility, dependability, and confirmability during data collection and analysis. **Results**: A thematic analysis revealed that, while psychia-trists and psychological counselors viewed anhedonia as a significant treatment target, nurses were more focused on immediate patient safety concerns. Participants recognized the potential of EFT training to alleviate anhedonia but identified several implementation challenges, including patient resistance, cognitive limitations, and the need for tailored interventions. **Conclusions**: The research results indicated that psychiatric nurses had relatively poor ability to identify anhedonia. Therefore, it is necessary to enhance the awareness of psychiatric nurses regarding the clinical significance of anhedonia, and incorporate knowledge related to anhedonia into routine nursing training. It is suggested that communication and collaboration among psychiatrists, psychiatric nurses and psychological counselors should be strengthened, and an assessment and feedback process for the lack of anhedonia in patients with MDD should be established, so as to assist these patients in achieving faster psychological recovery. Given the sufficient staffing conditions in the field of psychiatry nursing in China, the design concept and curriculum of EFT training for psychiatry nurses with a psychological therapist certificate should be promoted. Encourage psychiatry nurses with a psychological therapist certificate to conduct offline and online group EFT training intervention forms for MDD patients in the hospital wards during their hospitalization periods, as well as after discharge at home.

## 1. Introduction

Major depressive disorder (MDD), as a common mental disorder, has become a significant issue affecting mental health on a global scale [1]. According to the statistics from the World Health Organization, it is estimated that 3.8% of the population suffers from MDD [2], and the prevalence rate is still increasing year by year [3]. In China, the annual prevalence rate of MDD is 3.6%, the lifetime prevalence rate is 6.8%, and the total number of patients exceeds 90 million [4]. From the perspective of causing illness and disability, MDD has risen to the 12th position among global causes of disability in 2021, and it is one of the three diseases with the fastest growth rate [5]. Among all the diseases that cause loss of Years Lived with Disability (YLD), MDD ranks second, accounting for 6.2% of the total YLD [5].

Given the high prevalence and high disability rate of MDD, identifying and intervening in the key symptoms of MDD patients holds significant value. According to the Diagnostic and Statistical Manual of Mental Disorders, Fifth Edition (DSM-5), anhedonia and depressed mood are two of the core criteria for diagnosing MDD. Anhedonia refers to a marked reduction in interest or pleasure in activities. Treadway and Zald first proposed in 2011 that anhedonia is a multidimensional construct, comprising anticipatory, consummatory, and decisional anhedonia [6]. Based on the reward processing framework, Rømer et al. [7] further defined anhedonia as “a reduced ability to pursue, experience, and learn about pleasure”. Clinical surveys had shown that the proportion of patients with MDD experiencing a lack of anhedonia ranges from 30% to 75% [8,9,10]. In the subjective experience of MDD patients, anhedonia was regarded as the second biggest source of distress in life [11]. Anhedonia is a predictive factor for suicidal thoughts in adolescent individuals and psychiatric patients [12,13,14]. Meta-analysis showed that when the individual’s depression level was controlled, the association between anhedonia and suicidal ideation was significant [15]. A large-scale cohort study had shown that the improvement of anhedonia was the strongest predictor of the improvement in the psychological and social functions of patients with MDD [16]. After adjusting for the overall severity of MDD and other clinical covariates, anhedonia was still able to predict poor antidepressant treatment outcomes in patients with MDD [17]. Therefore, the symptom of anhedonia in MDD patients requires intensified intervention.

The significance of intervention for anhedonia has received increasing attention in recent years. The existing intervention methods (including medication, physical therapy, and traditional psychological therapy) have limitations in alleviating this symptom. In terms of drug treatment, selective serotonin reuptake inhibitors are widely used as the first-line treatment for MDD, but their effectiveness in addressing the symptom of anhedonia during treatment is not satisfactory [18]. The current mainstream psychological intervention methods (such as cognitive behavioral therapy, mindfulness therapy, behavioral activation therapy, etc.) have not achieved satisfactory results in improving the condition of anhedonia [19]. The possible reason may be that the mainstream psychological intervention methods mostly aim at suppressing negative emotions rather than enhancing the reward processing process or activating positive experiences. This might make it difficult to effectively alleviate anhedonia [20,21]. Episodic Future Thinking (EFT) training is an emerging training method that aims to help individuals construct positive future scenarios and solutions, thereby changing their negative expectations and emotional experiences regarding the future [22]. This intervention method, by stimulating individuals’ hope for the future and encouraging positive actions, may potentially have an effect in alleviating anhedonia [23]. EFT training enhances the availability of positive outcome representations in individuals, buffers reward discounting, and boosts positive expectations to promote the occurrence of beneficial behaviors [22]. The six intervention components of EFT training are valence, vividness, contextuality, future orientation, specificity, and intertemporal choices [24]. Hallford et al. [25] conducted an EFT training program for patients with MDD for a period of two weeks. The results showed that the participants’ anhedonia significantly improved, their level of behavioral activation was high, and their overall function improved. Bär et al. [26] employed the ecological momentary research method and conducted a 12-day EFT training for patients with depressive emotions. The results showed that the EFT intervention could stimulate the participants’ motivation to undertake a certain activity [26]. Bogaert et al. [27] conducted EFT training for undergraduate students with depressive mood in the Netherlands. The results showed that EFT training improved the participants’ mental imagery level, but the improvement in anhedonia was not significant. In China, Zhou et al. [28] conducted a 7-day EFT training for college students with depressive emotions. The results showed that the participants in the EFT training experienced a reduction in depression and anxiety, and an improvement in the vividness of positive imagery. Research on EFT training in China is still in its infancy and has not yet been conducted in a clinical setting for patients with MDD. The intervention of EFT training for patients with MDD requires understanding the views of different healthcare professionals (such as psychiatrists, psychiatric nurses, and psychological counselors) regarding EFT training. However, most of the existing research on EFT training focuses on the efficacy assessment for patients. The viewpoints of different healthcare professionals regarding EFT training have not yet been fully understood.

In the research on psychological intervention for MDD patients, psychological therapists and counselors are usually the main providers [29]. However, there is a shortage of psychological professionals in China. By the end of 2020, there were a total of 43,816 psychiatrists, 139,642 registered nurses, 4354 psychological therapists and 6302 psychological counselors working in mental health institutions across the country in China [30]. According to the human resource data comparison of the 2020 Mental Health Atlas report by WHO [31], the number of psychiatrists and registered nurses in China has exceeded the levels of middle- and high-income countries, while the number of psychological therapists and psychological counselors is significantly lower than that of middle- and high-income countries [30]. Against the policy background of documents issued by the National Health Commission, such as the Work Plan for Exploring Characteristic Services for the Prevention and Treatment of Depression [32] and the Notice on Launching the “Year of Pediatric and Mental Health Services” Initiative (2025–2027) [33], it is necessary to explore psychological intervention models involving multidisciplinary medical personnel. In China, with the reform of mental health policies and the advancement of nursing competencies, psychiatric nurses holding psychotherapist certification are legally permitted to conduct psychological interventions. The current regulations and certification system provide psychiatric nurses with legitimate qualifications for psychotherapy, enabling them to participate more extensively in psychological interventions for MDD. Previous studies have confirmed the effectiveness of nurse-led psychological interventions for patients with depressive symptoms [34,35,36]. However, few studies have explored how psychiatric nurses with psychotherapist certification attend to and intervene in anhedonia symptoms among patients with MDD. Therefore, this study used qualitative interviews to explore and compare the perspectives and previous coping strategies of psychiatrists, psychiatric nurses, and psychological counselors regarding anhedonia. In addition, the study examined their views and implementation suggestions on EFT training for MDD patients led by psychiatric nurses with psychotherapist certification.

## 2. Methods

### 2.1. Researcher Role

The research team consisted of doctoral students and professionals who had earned a Ph.D. in the fields of psychology and nursing. They had a solid academic foundation, good communication skills, and systematic training in qualitative research methods. All research team members had prior research experience in the field of psychiatric and mental health nursing and were familiar with the manifestations of anhedonia and related psychological interventions for patients with depression. No hierarchical or supervisory relationships existed between the researchers and the participants.

Before the study commenced, the researchers explicitly reflected on and documented their positionality and assumptions through written reflexive memos (e.g., recognizing that anhedonia might be an underappreciated issue in nurses’ clinical practice). Throughout the data collection and analysis processes, reflexive journals and bracketing techniques were employed to identify and manage potential personal biases. The research team held regular meetings to discuss how their prior experiences and perspectives might influence interview questions or data interpretation. During the analysis, the researchers repeatedly reviewed the original transcripts to ensure that the themes were inductively derived from participants’ narratives rather than shaped by researchers’ preconceptions.

### 2.2. Study Design

This study adopted a qualitative research design and conducted semi-structured interviews with psychiatrists, nurses, and psychological counselors from a tertiary public hospital in Wuhan, Hubei Province, China, to explore healthcare professionals’ (psychiatrists, nurses, and psychological counselors) attention to and previous coping strategies for anhedonia in patients with MDD. Furthermore, the study examined their views and future implementation suggestions regarding EFT training led by psychiatric nurses with psychotherapist certification in China.

### 2.3. Ethical Considerations

This study was approved by the Ethics Committee of Wuhan University (approval no. WHU-LFMD-IRB2024078). The participants signed a paper informed-consent form. To ensure the confidentiality of research data, all interview recordings were accessible only to members of the research team and were stored on encrypted hard drives, with no access permitted to external personnel. After transcription, identifiable information such as names was replaced with coded identifiers, and all potentially identifying details in the transcripts were removed or obscured. The anonymized transcripts were used solely for research analysis and did not contain or disclose any individual information.

### 2.4. Participants

This study used purposive sampling to recruit participants, selecting psychiatrists, psychiatric nurses, and psychological counselors from the psychiatric department of a tertiary general hospital in Wuhan, Hubei Province, China, for interviews. This hospital’s psychiatric department serves as a national regional medical center for mental disorders in China and has a broad patient base. This hospital’s psychiatric department possesses a well-established treatment system for MDD, experience in psychosocial interventions, and a favorable clinical research environment.

Participants were stratified by professional category (psychiatrists, nurses, and psychological counselors) and by years of work experience. These three professional groups hold core and complementary roles in the assessment, treatment, and psychological intervention of patients with MDD. Psychiatrists are primarily responsible for diagnosing MDD and providing pharmacological treatment. Psychiatric nurses maintain frequent contact with MDD patients through ward care and emotional support. Psychological counselors have professional experience in the specific implementation of psychological interventions for MDD patients. Joint interviews with these three groups facilitate a comprehensive understanding of the feasibility and potential barriers of EFT training in clinical practice.

The research team contacted eligible healthcare professionals and psychological counselors through the ward head nurses; all participants had long-term clinical experience in psychiatry. The sample size followed the data saturation principle of qualitative research, and recruitment ceased once no new themes emerged in consecutive interviews.

The inclusion criteria follow: holders of psychological therapist or counselor certificate, at least one year of experience in treating psychiatric patients, and voluntary participation and signed informed consent. The exclusion criteria were as follows: participants on leave for more than three months during the study, those who were studying abroad for more than three months, and those who were unwilling to participate.

The sample size followed the data saturation principle, meaning that saturation was reached when new data began to repeat previous data [37]. The research team conducted preliminary coding and topic organization after each two interviews were completed to monitor emerging concepts and themes. When no new coding or theme categories appeared in the interviews of three consecutive participants, and all themes were fully elaborated, it was considered that the topic saturation was reached. After the 13th respondent’s interview, the research team found during the data organization and preliminary coding process that no new themes or concepts emerged in the new interviews, only the existing themes were supplemented and refined. Then, two more respondents were interviewed to confirm that no new information appeared, and finally it was confirmed that the data had reached saturation. The judgment of data saturation was independently made by two researchers and reached a consensus in the team meeting.

### 2.5. Data Collection

Before the formal interview began, the interviewer introduced himself to the respondent and established a friendly and harmonious relationship with him. There was no direct management, superior-subordinate or clinical care relationship between the researcher and the respondent. The two interviewers were of the researcher’s identity (Minghao Pan and Huijing Zou), and did not undertake the respondents’ daily clinical work or performance evaluation duties. During the interview, the researcher maintained a neutral stance and avoided using guiding questions; for possible power/identity influences, the research team monitored and recorded them through reflection logs and team discussions.

Face-to-face interviews were conducted in a quiet office setting. The researcher explained the topic and purpose of the study to the participants and informed them that their interviews would be recorded. The participants were assured that their data would remain anonymous, and they signed the informed consent form. Participants first completed a demographic information survey that included basic sociodemographic information.

Based on a review of the domestic and international literature on psychological interventions for anhedonia in patients with MDD, a preliminary interview outline was developed (Version 1.0). To assess its clarity and relevance, a pilot interview was conducted with two psychiatric healthcare professionals (one psychiatrist and one psychiatric nurse, each with over five years of clinical experience). Feedback from the pilot indicated that some questions were repetitive or ambiguously worded. The interview guide was subsequently revised to improve question clarity and logical flow, resulting in the final version (Version 2.0), which was used in the formal interviews. The final interview guide is presented in Table 1 and provided in the Supplementary Material.

Interviews and content coding were conducted simultaneously by the two researchers (Minghao Pan and Huijing Zou), and the interview outline was dynamically adjusted during the process. Verbal and nonverbal expressions of the participants were observed and recorded. The average interview duration was approximately 32 min. Within 24 h of each interview, audio recordings were transcribed, resulting in approximately 100,000 valid words.

### 2.6. Data Analysis

Two researchers (Minghao Pan and Huijing Zou) conducted a manual review of the transcribed text, comparing the audio recording with the text word-by-word and sentence -by-sentence, and verifying with the respondents to ensure there were no omissions or misunderstandings.

This study followed Colaizzi’s phenomenological analysis method for data analysis [38]. The operational steps included: repeatedly reading all interview texts to familiarize with the data; extracting important statements related to the research topic; coding the important statements to form initial concepts; classifying and aggregating the initial concepts into themes; verifying the accuracy of the themes by constantly reviewing the original data; integrating the final themes to form research conclusions and confirming the understanding with the respondents through member verification when necessary; and ensuring the consistency of coding through independent arbitration by a third researcher (Dan Luo).

Throughout the analysis process, the research team maintained audit trails, documenting analysis decisions, the evolution of themes, and discussion results to ensure the transparency and traceability of the analysis process. In addition, the research team adopted data triangulation and researcher triangulation to enhance the reliability of the results. Researchers also consciously searched for negative cases, i.e., statements inconsistent or contrary to the main theme, to ensure a comprehensive understanding of the data and improve the reliability of the analysis. NVivo 12 Plus software was used to assist in text management, concept extraction, and theme classification.

## 3. Results

### 3.1. Demographic Data

A total of 15 healthcare professionals participated in the interviews, including 3 psychiatrists, 6 psychiatric nurses, and 6 psychological counselors or psychotherapists. The ages of the healthcare professionals ranged from 32 to 62 years old, and their working years were from 4 to 29 years. The experience of the healthcare professionals in providing psychological counseling or psychotherapy in the psychiatry department was 2 to 20 years. All participants worked in the psychiatry department for treating depression and had previously conducted group psychotherapy. The general information of the interviewees is shown in Table 2.

### 3.2. Interview Results

**Figure 1:**
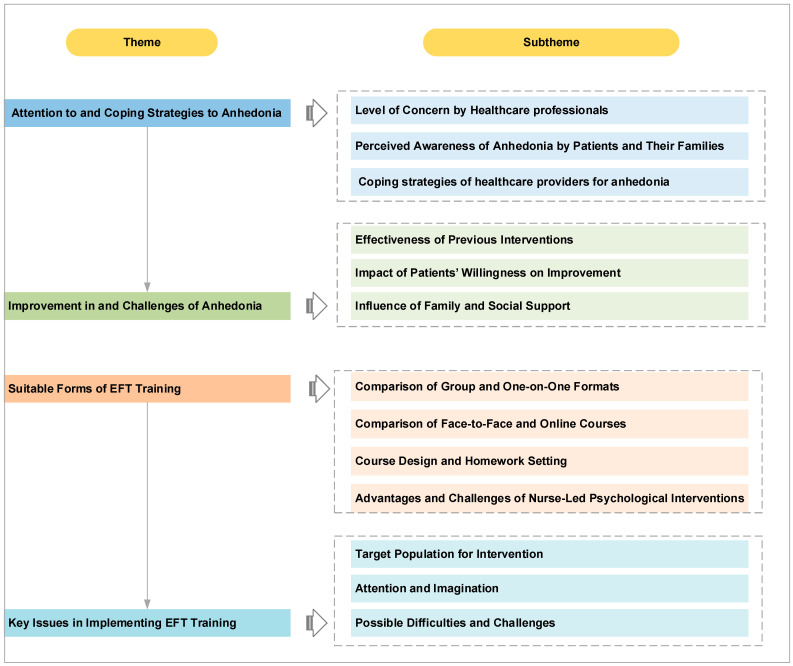
Themes and subthemes of participants’ views on anhedonia and EFT training.

The theme and subtheme models derived from the interviews are shown in Figure 1. The main themes included attention and responses to anhedonia, improvement and challenges of anhedonia, suitable forms of EFT training, and key issues in implementing EFT training.

#### 3.2.1. Attention to and Responses to Anhedonia

Attention to and responses to anhedonia were divided into three subthemes: the level of concern by healthcare professionals, perceived awareness of anhedonia by patients and their families, and coping strategies of healthcare providers for anhedonia.

##### Level of Concern by Healthcare Professionals

Three doctors interviewed demonstrated a high level of concern regarding the symptoms of anhedonia in patients with MDD. Five psychological counselors have also described that patients with MDD often experience such symptoms as loss of interest in previously enjoyable activities. Nurses, however, focused more on the risks of self-harm and suicide, although they also recognized loss of interest as an important issue.

Anhedonia is very common in depression patients; we have assessed over 1000 cases, and about 70–80% * of these patients exhibit this symptom.

(N8 Doctor)

*From my observation, anhedonia is quite severe in patients. When we assess or diagnose MDD, anhedonia is one of the key symptoms—patients often lose interest in many things*.

(N14 Psychological Counselor)

*When we conduct psychotherapy, the focus is more on uncovering the irrational beliefs. As for symptoms like anhedonia, they are common, but less often addressed directly*.

(N15 Psychotherapist)

*In clinical practice, we don’t assess anhedonia specifically. We may look at whether a patient has lost hope for the future, which overlaps with anhedonia. But our main focus remains on evaluating risk*.

(N5 Head Nurse)

* Note: These figures reflect clinicians’ subjective perceptions based on their professional experience, rather than epidemiological data.

##### Perceived Awareness of Anhedonia by Patients and Their Families

The two doctors and two nurses in the interview pointed out that many patients and their families exhibited poor ability in recognizing anhedonia. Four psychological counselors stated that many patients might have lost interest in activities that previously brought them joy but they did not realize how anhedonia was affecting their recovery.

*Many patients and families have poor awareness of anhedonia. This lack of understanding can hinder their recovery*.

(N5 Head Nurse)

*Many patients, including their families, fail to recognize that losing interest or hope for the future is actually anhedonia*.

(N6 Head Nurse)

*For patients with MDD, they may experience happy events but suppress their feelings, not acknowledging or enjoying the happiness*.

(N8 Doctor)

##### Coping Strategies of Healthcare Providers for Anhedonia

Three psychiatrists interviewed indicated that pharmacological treatment is the primary approach for addressing anhedonia in patients with MDD. Five psychotherapists interviewed reported providing psychological interventions tailored to patients’ interests and their own areas of expertise, such as hypnotherapy, mandala painting, and narrative therapy. The nurses interviewed reported taking relatively few measures to address anhedonia in patients with MDD.

*In clinical practice, we primarily use medication. After stabilizing the patient’s condition, we provide some psychological counseling and interventions*.

(N8 Doctor)

*I use hypnosis therapy, which is quite different from standard psychotherapy*.

(N11Psychological Counselor)

*I use narrative therapy to understand when and why the patient lost their ability to experience pleasure*.

(N14 Psychological Counselor)

#### 3.2.2. Improvement in and Challenges of Anhedonia

This theme was divided into three subthemes: the effectiveness of previous interventions, the impact of patients’ willingness to improve, and the influence of family and social support.

##### Effectiveness of Previous Interventions

Previous interventions have shown limited effectiveness in improving the symptoms of anhedonia. Moreover, the effectiveness of these interventions varies between individuals. The four psychological counselors and three doctors generally believe that improving anhedonia is a long-term and complex process. Medication may control symptoms in the short-term; however, long-term improvement requires cognitive shifts and increased support.

*The improvement is modest—on a scale where 0 is neutral, patients might score 4 or 5, indicating a slight sense of pleasure*.

(N14 Psychological Counselor)

*The effects are variable. If a patient has strong family support, they may recover from anhedonia more effectively, but about 1/3 to half of patients will still have residual symptoms*.

(N8 Doctor)

*Medication can control symptoms in the short term, but long-term improvement requires a shift in cognition*.

(N14 Psychological Counselor)

##### Impact of Patients’ Willingness on Improvement

Patients’ willingness to engage with treatment plays a crucial role in improving anhedonia. The six psychological counselors emphasize that patients’ cooperation and motivation directly influence treatment outcomes, including adjusting treatment frequency, overcoming cognitive rigidity, and ensuring treatment progress. If a patient is unwilling to participate, their progress may be slow or limited. Conversely, patients with strong motivation are more likely to actively engage in treatment and experience faster improvement.

*Whether any treatment succeeds depends on the patient’s willingness to cooperate and their mental flexibility*.

(N15 Psychotherapist)

*It depends on the individual’s willingness. If they have a strong desire to change, improvement can happen quickly. Otherwise, the outcome is poor*.

(N12Psychological Counselor)

*Patients with strong motivation improve more quickly. However, most depression patients have low energy and are resistant to change, so their progress is slower*.

(N12 Psychological Counselor)

##### Influence of Family and Social Support

Family and social support play significant roles in alleviating anhedonia in patients with MDD. The four psychological counselors in the interview pointed out that patients often feel empty and lose interest in certain things. The two doctors and four nurses in the interview also believed that family and social support, understanding, and assistance could provide the necessary emotional comfort for patients with MDD.

*Patients with MDD feel very empty inside, and family support helps improve their condition over time*.

(N13 Psychological Counselor)

*Family and social support can facilitate recovery*.

(N8 Doctor)

*Family support, social support; that is conducive to his condition*.

(N10Psychological Counselor)

*Some patients are hospitalized for MDD, but if their family doesn’t acknowledge the problem, their recovery is slower. Even after recovery, returning to a non-supportive family environment can destabilize their condition*.

(N8 Doctor)

#### 3.2.3. Suitable Forms of EFT Training

Suitable forms of EFT training include four subthemes: a comparison between group and one-on-one formats, comparison between face-to-face and online courses, course design and homework setting, and the advantages and challenges of nurse-led psychological interventions.

##### Comparison of Group and One-on-One Formats

The four psychological counselors interviewed pointed out that both group and one-on-one therapy have advantages and disadvantages. Group therapy allows a greater number of individuals to be treated in the same amount of time, thus improving overall treatment efficiency. In addition, group therapy provides a relatively realistic social environment in which individuals can learn how to interact with others, helping them adapt better to real-life situations. However, in a group setting, individuals may be less willing to discuss deeper issues because of privacy concerns, which can affect treatment outcomes.

*The advantage of group therapy is that it improves efficiency, and it simulates a relatively real environment*.

(N15 Psychotherapist)

*Group therapy is generally more difficult because many patients here have emotional problems*.

(N15 Psychotherapist)

*I think the effect of group therapy should be similar to one-on-one, as long as the group therapy is conducted continuously according to the treatment plan*.

(N12Psychological Counselor)

*In a group setting, everyone listens, but they may not be very open to discussing deeper personal issues, as some things need to be kept confidential*.

(N10Psychological Counselor)

##### Comparison of Face-to-Face and Online Courses

The two doctors, four nurses and five psychological counselor interviewed all stated that indicated that given patients’ hospitalization periods and individual characteristics, psychological interventions have gradually shifted from emphasizing face-to-face treatment to online forms, which are now widely accepted. Especially since the COVID-19 pandemic, therapists have adopted face-to-face sessions to establish trust, followed by online sessions for remote psychological interventions.

*Online psychological therapy is possible, as long as the patient cooperates. During the pandemic, for example, if the therapist couldn’t come or if patients couldn’t go out, the therapist would add them on WeChat and conduct video consultations*.

(N2 Nurse)

*I think online courses are feasible. Some patients find it inconvenient to come to the hospital, so they receive online consultations and discuss their symptoms online, and they are quite willing to cooperate*.

(N8 Doctor)

*Previously, face-to-face consultations were emphasized, where one-on-one interaction allowed for judgment based on the patient’s expressions and tone. However, for depression patients, it’s difficult to get them to come for consultations because they don’t want to leave their homes. Online interventions can still be effective*.

(N12Psychological Counselor)

*Generally speaking, since our patients are hospitalized for short periods, it’s fine to conduct the later sessions online, as long as trust is established with the patient in the early stages*.

(N13Psychological Counselor)

##### Course Design and Homework Setting

Most respondents believed that the frequency, cycle, and duration of the course should be designed according to patients’ hospitalization schedules and duration of stay. The four nurses who were interviewed suggested that the course content should be engaging, focusing on a patient’s psychological state and interests to stimulate their willingness to express themselves. The four psychological counselors have pointed out that the course arrangement should be based on the patient’s specific condition and severity; for mild cases, 4–6 sessions would be sufficient for resolving most issues.

*I think, based on the hospitalization period, a routine frequency of 1–2 sessions per week and a total of 4–6 sessions would be appropriate*.

(N3 Nurse)

*Depressed patients generally have less desire to express themselves; some may not want to speak. Our group therapy sessions typically last 50 min to an hour*.

(N2 Nurse)

*I believe that unless the content is extremely engaging and stimulates patients’ willingness to express themselves, the course may feel short or too long, and the patient may lose interest*.

(N3 Nurse)

*For mild patients, 4–6 sessions can be very effective. It can address urgent issues and provide immediate relief*.

(N10Psychological Counselor)

*Here, patients usually have short hospitalization times, so about three sessions of psychological therapy is typical, while more severe cases might require about four*.

(N15 Psychotherapist)

Psychological counselors also emphasized that homework should not be viewed as “assignments” but as suggestions or exercises. Homework should consider a patient’s self-discipline and motivation and include gentle reminders. Patients should not be forced to complete it and their progress should be discussed in the next session to encourage their continued participation and progress. Nurses also emphasized the issue of low motivation and lack of engagement with homework in patients with depression.

*We will remind patients about homework repeatedly, but we shouldn’t treat it as an assignment. If it’s treated as an assignment, they may refuse to do it*.

(N12 Psychological Counselor)

*We generally suggest [that] the patient do the homework, but we don’t force them. Next time, we’ll discuss their progress with them*.

(N13Psychologist)

*For our patients, homework is not highly valued, as depression patients often have low motivation*.

(N1 Nurse)

##### Advantages and Challenges of Nurse-Led Psychological Interventions

Most psychological counselors and nurses agreed that psychological interventions led by nurses with psychological therapist certification were feasible. The three psychological counselors have pointed out that the key to nurse-led interventions is finding a balance between compassion and professionalism. The three nurses in the interview stated that in terms of clinical practice, for qualified nurses to lead psychological intervention, they need to possess excellent communication skills, rich experience, professional theoretical knowledge, and corresponding qualifications. Furthermore, when nurses lead interventions, they may be affected by their emotions and may need professional support to maintain their emotional well-being.

*Nurse-led interventions are definitely effective. Some patients may not seek help from psychological professionals, but instead go to doctors or nurses*.

(N11Psychological Counselor)

*In clinical practice, if nurses have good communication skills, rich experience, professional knowledge, and qualifications, they can lead psychological interventions*.

(N1 Nurse)

*When nurses lead psychological interventions, they need to find a balance between compassion and professionalism. The most important ethical boundary is having a conscience and compassion. Technical knowledge is also crucial, as they need to understand the patient’s needs and how to address them*.

(N11Psychological Counselor)

*Nurses leading psychological interventions may experience emotional and cognitive challenges. They need time to adjust. For example, nurses who provide psychological therapy in our department receive regular professional counseling to maintain emotional stability*.

(N5 Head Nurse)

#### 3.2.4. Key Issues in Implementing EFT Training

The key issues in implementing EFT training are divided into three subthemes: the target population for intervention, attention and imagination, and possible difficulties and challenges.

##### Target Population for Intervention

Healthcare professionals who were interviewed indicated that a patient’s current state must be considered. Patients with severe depression or strong self-harm tendencies may require psychological crisis intervention. The two nurses and three psychological counselors in the interview pointed out that older patients may be more stubborn, less cooperative, and impatient during treatment. Therefore, being mindful of the tone and language used during psychological interventions is important. The four nurses pointed out that in clinical practice, interventions are often aimed at patients who have stabilized emotionally after admission.

*We should add exclusion criteria during the screening process, such as severely negative or suicidal patients, or those on heavy psychiatric medications*.

(N5 Head Nurse)

*You can choose younger patients for intervention, as they are more cooperative. Older patients may become impatient and less cooperative during treatment*.

(N2 Nurse)

*Patients should be selected based on their condition, focusing on those whose emotions are stable*.

(N1 Nurse)

##### Attention and Imagination

The three psychological counselors and two doctors have emphasized the importance of attention and imagination in EFT training for patients with MDD. The primary challenge with this training involves the ability of patients with MDD to focus and imagine. Attention is the foundation of training; without it, treatment will be difficult. Furthermore, as EFT involves the construction of future scenarios, the patient’s imagination is crucial. Patients with MDD often have low energy and slow thinking abilities, which make it difficult for them to imagine specific scenarios independently. Before beginning the training, patients should be guided to develop their imagination for the training to be effective.

*Attention is a big problem for patients, and their ability to imagine is also crucial. Since this training involves constructing future scenarios, you need to first build their ability to imagine before the training can lead to significant improvement*.

(N8 Doctor)

*This requires engaging the patient’s attention, especially their imagination*.

(N13Psychological Counselor)

*We generally assess patients with MDD in two ways: first, their attention, as without it they will not be able to continue the training. Second, we assess their imagination. Many patients with MDD find it difficult to imagine. If their attention and imagination are both good, they will definitely be able to learn. If either is lacking, it will be difficult for them to imagine*.

(N12Psychological Counselor)

##### Possible Difficulties and Challenges

The four psychological counselors who participated in the interviews pointed out that establishing a good therapeutic relationship was crucial for the effectiveness of treatment. Some patients may lack initiative and attend only because of parental pressure, which can lead to poor treatment outcomes. Some nurses indicated that patients may think of negative things during the imaginative process, which can lead to emotional distress. In such cases, a professional therapist should be present to guide the patients. The three nurses also mentioned that during psychological interventions, their emotions could be influenced by the patients, and experienced psychotherapists are needed to help guide and stabilize nurses’ emotions to ensure smooth progress of the training. Member turnover during group therapy can also affect treatment outcomes. Fixed groups help increase feelings of safety, thereby enhancing therapeutic effects. Moreover, some patients may take a long time to integrate into a group and be unwilling to cooperate, leading to outbreaks of depressive symptoms that are difficult to control. These situations may occur during treatment.

*Some of these patients are not very cooperative; some come just because their parents asked them to. They come to chat, and you could do 10 sessions with them, but I think it won’t be of much use. If you want this to be effective, the first thing you need is a good therapeutic relationship; this is extremely important*.

(N15 Psychotherapist)

*During the imagination process, they might think of some negative things. So when guiding them, it is essential to have a professional psychological counselor nearby. If the patient suddenly experiences negative emotions, they can help bring the patient back*.

(N2 Nurse)

*People feel safer in familiar environments. If the members are more fixed, there’s an established rapport and trust. With trust comes safety, and only then can they fully invest in the treatment*.

(N14 Psychological Counselor)

*Some people are slow to warm up, and it may take a long time for them to integrate. Others may be unwilling to cooperate*.

(N15 Psychotherapist)

## 4. Discussion

This study conducted qualitative interviews to understand and compare the attention paid by psychiatrists, psychiatric nurses, and psychological counselors to anhedonia, as well as their previous coping methods. Additionally, this study explored the perspectives and implementation suggestions of psychiatrists, psychiatric nurses, and psychological counselors regarding the EFT training led by psychiatric nurses with a psychotherapy certificate.

This study revealed that different healthcare professionals have varying levels of attention to the issue of anhedonia. The psychiatrists and psychological counselors interviewed in this study regarded anhedonia as a key point for diagnosis and treatment, while the nurses interviewed focused on the risk of self-harm and suicide among patients with MDD [39]. Nurses usually receive more training in clinical practice regarding the identification of self-harm, suicide risks and safety plans. Therefore, psychiatric nurses tend to prioritize the identification and handling of safety risks in their routine nursing duties [40,41]. However, anhedonia, as one of the core dimensions of depression, has been proven to be highly correlated with the severity of depression, poor response to antidepressant treatment, and suicidal ideation [42]. Given that our interviews revealed that psychiatric nurses have relatively weak ability to identify anhedonia, efforts are needed to enhance the awareness of psychiatric nurses regarding the clinical significance of anhedonia and incorporate it into regular training. It is suggested that knowledge training for psychiatric nurses (such as understanding the common manifestations of anhedonia in patients with MDD, using anhedonia screening tools, and comprehending the assessment criteria for anhedonia) should be provided. It is recommended that communication and collaboration among psychiatrists, nurses, and psychological counselors should be strengthened, and an assessment and feedback process for anhedonia in MDD patients should be established, thereby assisting MDD patients in recovering more quickly.

The interview results showed that psychiatrists, psychiatric nurses and psychological counselors expressed their approval of the improvement concepts of EFT training. The psychiatrists and psychological counselors who participated in the interview, based on their own professional experience, identified the possible challenges that may arise when implementing EFT. For instance, patients with MDD who have severe depression and a tendency towards self-harm or suicide may experience significant emotional fluctuations during the reconstruction of thought scenarios [43]. For instance, patients with MDD suffer from problems such as distraction and limited imagination. Therefore, when conducting EFT training in clinical practice in China in the future, it is necessary to design EFT training course content and training methods that are adapted to the cognitive characteristics of MDD patients, and gradually stimulate the interest and participation of MDD patients. Psychiatrists and psychological counselors who participated in the interviews, based on their own experience, pointed out that for MDD patients in the ward, the treatment cycle and previous intervention forms should be taken into account to propose the intervention form of offline group therapy and online therapy at home after discharge for EFT training. The group therapy form has the advantages of being able to cover more patients simultaneously, simulating a real social interaction environment, and helping patients learn to express in a group setting [44]. Previous studies on EFT training for patients have mostly adopted a group format and have shown good acceptance. The Chinese population is characterized by introversion and a defensive attitude towards unfamiliar events from the outside. Therefore, when conducting EFT training in clinical practice in China in the future, the initial courses can be conducted in a face-to-face offline format to establish trust in team treatment, and the later courses can adopt a more convenient online format for EFT training. Previous studies have also pointed out that the online psychological intervention form can effectively supplement the psychological treatment for patients after discharge and provide continuous intervention after the hospitalization period ends [45].

The psychiatrists and psychological counselors who participated in the interviews in this study indicated that the EFT training led by psychiatric nurses with a license as psychotherapists has unique potentials and challenges. Compared with psychiatrists and Psychological Counselors, psychiatric nurses in the ward maintain frequent interactive nursing operations with MDD patients [46]. Psychiatric nurses can observe the emotional changes and behavioral manifestations of patients more promptly [46]. Moreover, psychiatric nurses have better experience in supportive communication, emotional companionship, and maintenance of treatment compliance for patients [47]. These professional capabilities formed by psychiatric nurses in clinical nursing provide strong support for implementing EFT training led by nurses for MDD patients in the ward. Currently in China, psychiatric nurses need to obtain the national qualification of a psychotherapist in order to carry out psychological intervention programs for patients in hospital wards. However, most of the psychiatric nurses with a psychotherapist certificate currently are mainly engaged in traditional psychological intervention programs such as CBT and DBT. For such new psychological intervention programs that involve imagination guidance, such as scenario construction and mental imagery, these nurses have less systematic learning. Given the sufficient staffing of psychiatric nurses in China, it is necessary to promote the design concept and course setting of EFT training for psychiatric nurses with a psychotherapist certificate to delve deeper into their learning. Encourage psychiatric nurses with a psychotherapist certificate to conduct offline and online group therapy EFT training intervention forms for inpatients with MDD in the wards and after discharge. At the same time, when these nurses with a psychotherapist certificate conduct EFT training in the early stage, they need to be accompanied by professional counselors with experience in group psychological intervention to supervise the teaching of each course. Therefore, in future practice, it is advisable to prioritize promoting the role of psychiatric nurses with a psychotherapist certificate in the hospital wards to organize and lead EFT training, and through continuous education and supervision, enhance their professional competence in the field of psychological intervention. This model not only helps alleviate the shortage of mental health therapists in psychiatry, but also provides a new direction for integrating psychological social intervention into clinical nursing work.

### Limitations

Although this study achieved thematic saturation in data analysis, it still has several limitations. Firstly, this study employed purposive sampling, and the interview participants were all psychiatrists, nurses, and psychological counselors from a public tertiary-level hospital in a provincial capital city in central China. Although the sample included in this study was stratified by the three categories of psychiatrists, nurses, and psychological counselors, as well as their years of work, it still failed to cover other potential influencing factors. For example, the stratification of the commonly used psychological intervention methods by doctors, nurses, and psychological counselors. Secondly, this study recruited participants from the psychiatry department of a single hospital. This hospital’s psychiatry department is a national-level regional medical center for mental diseases and has a wide range of patient sources. The research results may not be applicable to other levels of medical institutions. Thirdly, this study did not include the relevant viewpoints of patients with depression. This may have led to the research results mainly reflecting the viewpoints and practical experiences of medical professionals, rather than fully reflecting the views of MDD patients themselves on the absence of pleasure and the EFT training led by psychiatrists with psychological therapist certificates under the guidance of nurses.

## 5. Conclusions

This study indicates the need to enhance psychiatric nurses’ attention to anhedonia in patients with MDD. Subsequent expert consultations are needed to develop EFT training interventions for MDD patients led by psychiatric nurses with psychotherapist certification. Future research could explore the feasibility of such nurse-led EFT training for MDD patients and conduct cross-cultural validation to provide a basis for the broader implementation of clinical psychological nursing.

## Figures and Tables

**Table 1:** Interview outline.

Item	Question
1	How much attention do you pay to anhedonia symptoms in patients with MDD during treatment?
2	In your opinion, how effective is the treatment for anhedonia symptoms in patients with MDD?
3	Which medications or psychological treatments do you think are effective in improving anhedonia?
4	What form of episodic future thinking (EFT) training do you think is most suitable? Why?
5	How long do you think each session should last?
6	What do you think the frequency of these sessions should be?

**Table 2:** General information regarding participating psychiatrists, nurses, and psychological counselors.

Participant	Gender	Age (Years)	Professional Title	Position	Education	Years of Work Experience	Years of Experience in Psychological Counseling/ Psychotherapy
N1	Female	33	Supervisor Nurse/Intermediate Psychotherapist	None	Bachelor	9	2
N2	Female	34	Supervisor Nurse/Intermediate Psychotherapist	None	Bachelor	11	2
N3	Female	32	Supervisor Nurse/Intermediate Psychotherapist	None	Bachelor	8	2
N4	Male	38	Supervisor Nurse/Intermediate Psychotherapist	Head Nurse	Master	18	3
N5	Female	42	Supervisor Nurse/Intermediate Psychotherapist	Head Nurse	Master	22	2
N6	Female	48	Associate Chief Nurse/Intermediate Psychotherapist	Head Nurse	Master	29	4
N7	Female	47	Attending Physician/Intermediate Psychotherapist	None	Bachelor	18	3
N8	Male	34	Attending Physician/Intermediate Psychotherapist	None	Ph.D.	10	2
N9	Female	38	Associate Chief Physician/Intermediate Psychotherapist	Deputy Chief	Ph.D.	7	5
N10	Female	62	Level-2 Psychological Counselor	None	Associate Degree	15	8
N11	Female	43	Level-2 Psychological Counselor	None	Bachelor	9	9
N12	Male	62	Level-2 Psychological Counselor	None	Bachelor	27	20
N13	Female	36	Level-2 Psychological Counselor	None	Bachelor	9	9
N14	Male	32	Level-2 Psychological Counselor	None	Bachelor	7	7
N15	Female	33	Intermediate Psychotherapist	None	Ph.D.	4	4

Note: In Chinese medical institutions, there are two main types of certified professionals who may provide psychological interventions: Psychotherapist: This includes psychiatrists or assistant physicians in psychiatry who have completed standardized training in psychological therapy, as well as health professionals who have passed the national Health Professional Technical Qualification Examination in the specialty of psychological therapy and obtained the corresponding professional technical qualification. Titles such as Intermediate Psychotherapist reflect the level of training and clinical competence within this national framework. Psychological counselors: Individuals who have obtained the corresponding certification from the Chinese Psychological Society (CPS), which qualifies them to provide counseling services but does not automatically confer the ability to deliver clinical psychological therapy in medical settings. Titles such as Level-2 Counselor indicate the certification level within the CPS system.

## Data Availability

Our data were collected for the research group and are not publicly available. The datasets used and/or analysed during the current study are available from the corresponding author on reasonable request.

## Supplementary Materials

The following supporting information can be downloaded at: https://www.mdpi.com/article/10.3390/nursrep15110384/s1. Interview Guide (Version 2.0).

## Public Involvement Statement

There was no public involvement in any aspect of this research.

## Guidelines and Standards Statement

This manuscript was drafted against the COREQ (COnsolidated criteria for REporting Qualitative research).
