# Supplementary material for: Healthcare Professionals’ Perceptions of Anhedonia in Major Depressive Disorder and the Possibilities of Episodic Future Thinking Training: A Qualitative Study in a Tertiary Care Hospital
Source: Nurs Rep. 2025 Oct 28;15(11):384. doi: 10.3390/nursrep15110384 (PMC12655409; doi:10.3390/nursrep15110384)
Supplement: Supplementary file 1 [file nursrep-15-00384-s001.zip › nursrep-3862278-supplementary.pdf]

Supplementary Material – Interview Guide (Version 2.0)

1. How much attention do you pay to anhedonia symptoms in patients with MDD during treatment?

Follow-up probes:

1) How do you usually identify anhedonia in clinical practice?
2) Do patients or their families express concern about anhedonia?

2. In your opinion, how effective are current treatments for anhedonia symptoms in MDD?

Follow-up probes:

1) What challenges do you encounter in improving these symptoms?
2) How do patients respond to current treatment approaches?

3. Which medications or psychological treatments do you think are most effective in improving anhedonia?

Follow-up probes:

1) Could you share examples of what has worked or not worked in your experience?

4. What form of episodic future thinking (EFT) training do you think is most suitable for patients with MDD?

Follow-up probes:

1) Do you think group or individual sessions would be more effective?
2) How do you think patients would respond to this type of intervention?

5. How long do you think each session should last? What do you think the frequency of these sessions should be?

Follow-up probe:

1) What do you think would help patients maintain engagement over time?

6. Do you think it would be feasible for psychiatric nurses to deliver EFT-based interventions for patients with MDD?

Follow-up probes:

1) What advantages or challenges might arise from nurse-led psychological interventions?
2) What kind of training or support do you think nurses would need to implement EFT effectively?
